# Regeneration of Limb Joints in the Axolotl (*Ambystoma mexicanum*)

**DOI:** 10.1371/journal.pone.0050615

**Published:** 2012-11-21

**Authors:** Jangwoo Lee, David M. Gardiner

**Affiliations:** 1 Department of Developmental and Cell Biology, University of California Irvine, Irvine, California, United States of America; 2 The Developmental Biology Center, University of California Irvine, Irvine, California, United States of America; University of Liverpool, United Kingdom

## Abstract

In spite of numerous investigations of regenerating salamander limbs, little attention has been paid to the details of how joints are reformed. An understanding of the process and mechanisms of joint regeneration in this model system for tetrapod limb regeneration would provide insights into developing novel therapies for inducing joint regeneration in humans. To this end, we have used the axolotl (Mexican Salamander) model of limb regeneration to describe the morphology and the expression patterns of marker genes during joint regeneration in response to limb amputation. These data are consistent with the hypothesis that the mechanisms of joint formation whether it be development or regeneration are conserved. We also have determined that defects in the epiphyseal region of both forelimbs and hind limbs in the axolotl are regenerated only when the defect is small. As is the case with defects in the diaphysis, there is a critical size above which the endogenous regenerative response is not sufficient to regenerate the joint. This non-regenerative response in an animal that has the ability to regenerate perfectly provides the opportunity to screen for the signaling pathways to induce regeneration of articular cartilage and joints.

## Introduction

Many different approaches utilizing a variety of model systems have attempted to regenerate joint structures. Most of these efforts have focused on engineering specific joint tissues, articular cartilage in particular, that can be used for grafting to repair damaged joints. These efforts have been limited by the reality that cartilage has a limited endogenous regenerative response and forms fibrocartilage (scar tissue) in the joint in response to injury (see [1]). We already know from studies of salamanders that tetrapod limb joints in fact can regenerate perfectly during regeneration of an amputated limb (see [2], [3]). In addition, surgical defects to the articular cartilage of the axolotl (Mexican Salamander) knee joint made by resection of the medial femoral condyle to the level of the metaphysis regenerate intrinsically [4]. Thus the intrinsic regenerative response of the axolotl provides an opportunity to discover the mechanisms for inducing repair and regeneration of articular cartilage and joints.

Although development of limb joints has been studied extensively, very little is know about the regeneration of joints. Given the conservation of mechanism for development of tetrapod limbs, it is reasonable to assume that axolotl limb joint development is regulated by the same mechanisms as in more widely studied model systems such as the chick and mouse (see [5], [6]). Given the conserved morphology of tetrapod limb joints, along with the observation that a regenerated joint is morphologically the same as the joint that develops in the larva, it also is reasonable to assume that the mechanisms of joint development and regeneration are conserved. It is important to test the extent to which these assumptions are correct in order to justify utilizing the axolotl regeneration model system to provide insights for inducing repair and regeneration of joints in humans.

The global skeletal pattern of regenerating limbs has been analyzed repeatedly to draw conclusions about the mechanisms controlling pattern formation (see [7], [8]); however, little has been published regarding the details of the anatomy of either uninjured or regenerating joints in salamander limbs. The basic anatomy of axolotl joints with apposed articular surfaces between adjacent long bones that are encapsulated by connective tissues is very similar to mammals [4], [5], [9]. The expression patterns of the relatively few marker genes for mature joints that have been analyzed in the axolotl also are comparable to those in mammalian synovial joints [4], [9]. At the same time, some of the joints (e.g. knee) are different in the axolotl in that the synovial cavity is filled with fibro-cellular tissue rather than acellular synovial fluid as in the typical diarthrodial mammalian joint [4], [5], [9]. The possible function of these synovial cells is unknown, though when grafted into a skeletal defect in the diaphysis, they can participate in a regenerative response and appear to differentiate as both chondrocytes and synovial cells [9].

In spite of the ability to regenerate entire amputated limbs, including joints, there are injuries to the limb skeleton of axolotls that fail to regenerate. As in mammals, a skeletal defect that exceeds a critical size (CSD, critical size defect) is not regenerated in axolotls [10], [11], [12]. In both axolotls and mammals there is a localized chondrogenic response that results in callus formation, but this healing response is not adequate to regenerate the defect. In contrast to defects in the diaphyseal region, axolotls and mammals exhibit different responses to injuries to the articular cartilage and the epiphysis of the knee joint [4]. In mammals, injury to the epiphysis results in formation of fibrocartilage rather than regeneration of articular cartilage (see [1]). Similar injuries in the axolotl knee joint are repaired by regeneration of the defect [4]. One of the goals of the current study was to further characterize this intrinsic ability of axolotls (and presumably other salamanders) to regenerate surgical defects in the joint region.

**Figure 1:**
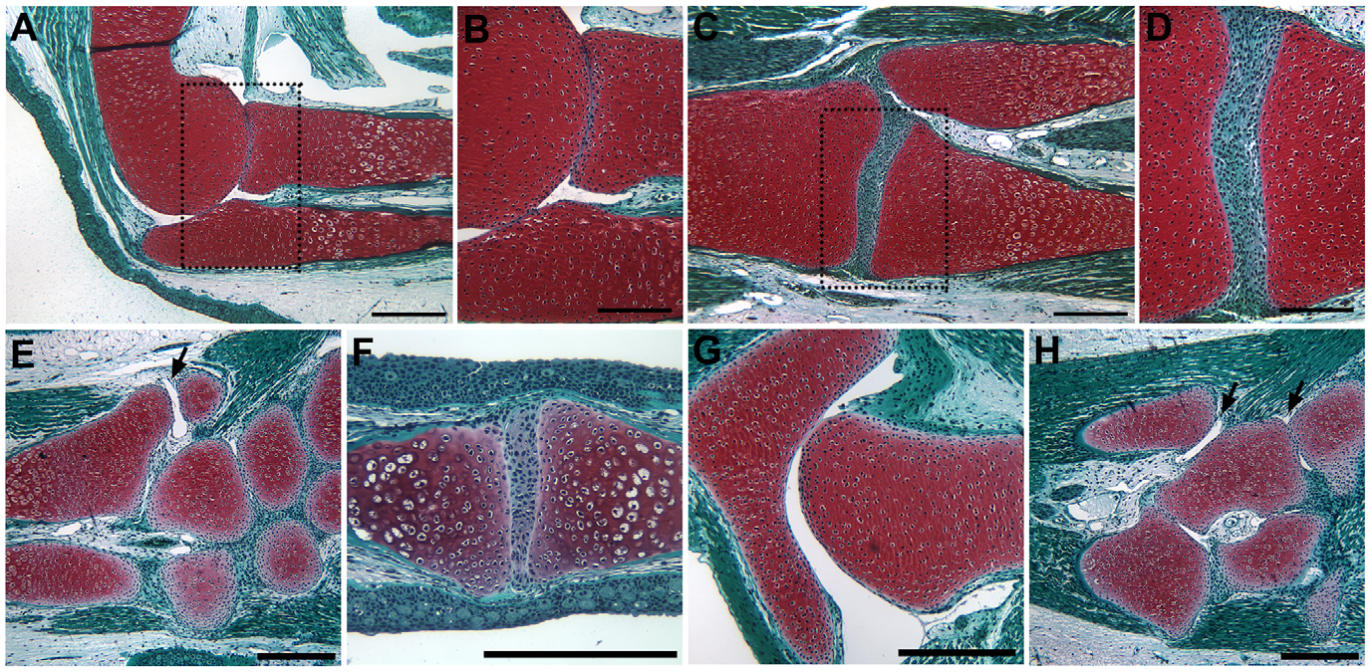
Joints in the forelimb and hind limb of the axolotl. The elbow joint was comparable to a mammalian synovial joint and lacked fibro-cellular tissue in the synovial cavity (A, B). Although the basic anatomy of the knee joint was similar to mammalian synovial joints, it differed in that the synovial space was filled with fibro-cellular tissues (C, D). The wrist (E) and ankle (H) joints exhibited a mixed phenotype in that the synovial space between some bones was fibro-cellular, but was acellular at other articulations (arrows in E and H). The interphalangeal joints of the fingers (E) and toes (not illustrated) were fibro-cellular; whereas, the hip (G) and shoulder (not illustrated) joints were acellular. Tissue sections were stained with Fast Green/Safranin O/Weigert’s Iron Hematoxylin. Boxed areas in (A) and (C) are illustrated at higher magnification in (B) and (C) respectively. The conserved anatomy was confirmed by analysis of the joints in two different animals. Scale bars in (A, C, E, G and H) = 500 µm; (B, D and F) = 200 µm.

In this paper, we describe the morphology and the expression patterns of marker genes during joint regeneration in response to limb amputation. These data are consistent with the hypothesis that the mechanisms of joint formation whether it be development or regeneration are conserved. We also have determined that defects in the epiphyseal region of both forelimbs and hind limbs in the axolotl are regenerated only when the defect is small. Thus, as is the case with defects in the diaphysis, there is a critical size above which the endogenous regenerative response is not sufficient to regenerate the joint. Since axolotl joints can regenerate perfectly in response to signaling associated with limb amputation, a non-regenerative CSD joint excision provides the opportunity to screen for the signaling pathways that control the regeneration of articular cartilage and joints.

**Figure 2:**
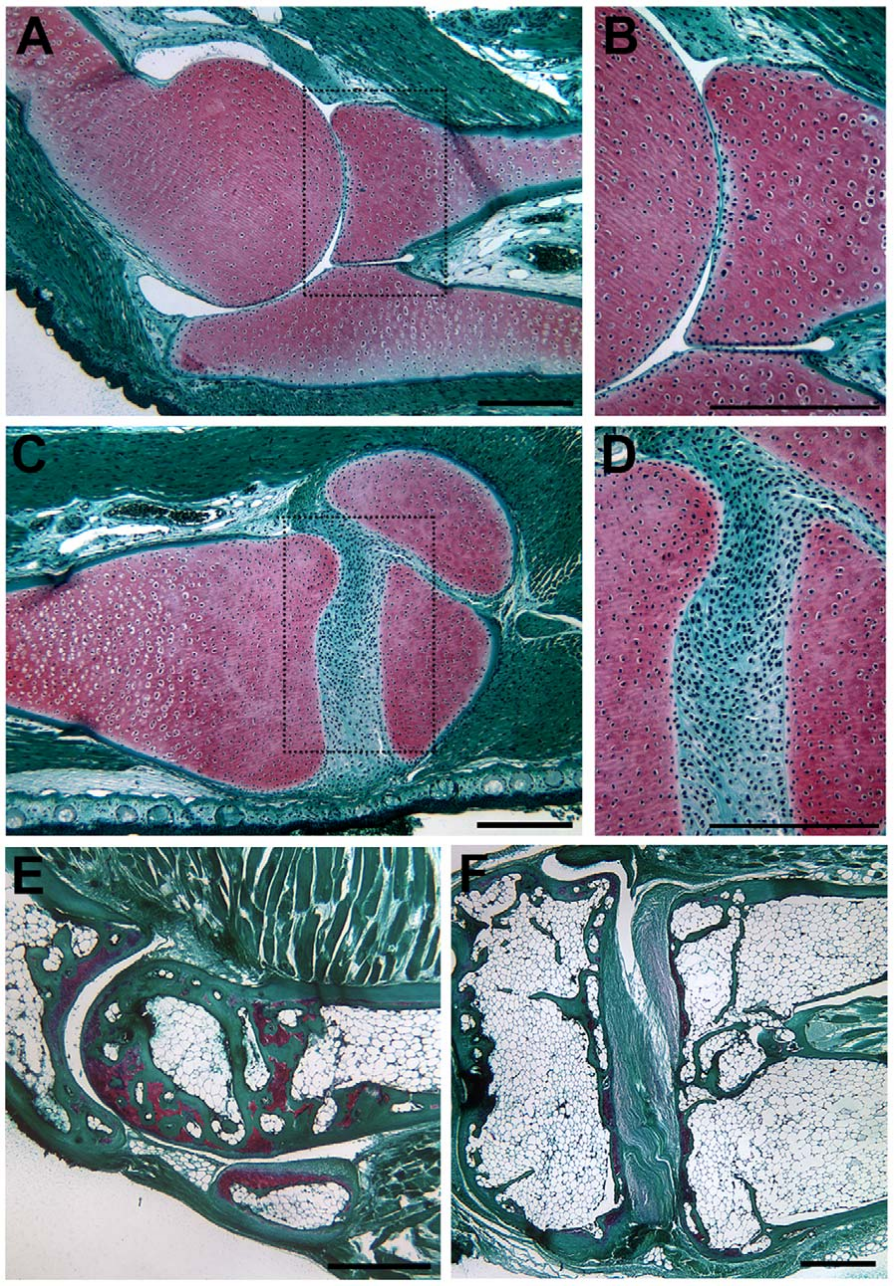
Joints in the forelimb and hind limb of the post-metamorphic axolotl and frog (*Xenopus tropicalis*). The elbow joint (A, B) and knee joint (C, D) of a post-metamorphic axolotl (treated with thyroid hormone to induce metamorphosis) were morphological the same as in neotenous adult axolotls (compare to Fig. 1 A–D). In adult *X. tropicalis*, the synovial cavity of the elbow joint was acellular (E); whereas, and in the knee the synovial cavity contained fibro-cellular tissue (F). This is the same pattern as observed in axolotl elbow and knee joints (compare to Fig. 1 A–DFig. 1 A–D; Fig. 2 A–D). Tissue sections were stained with Fast Green/Safranin O/Weigert’s Iron Hematoxylin. Boxed areas in (A) and (C) are illustrated at higher magnification in (B) and (C) respectively. The conserved anatomy was confirmed by analysis of the joints in two different post-metamorphic axolotls and two individual *X. tropicalis*. Scale bars = 500 µm.

## Materials and Methods

### Ethics Statement

This study was carried out in strict accordance with the recommendations in the Guide for the Care and Use of Laboratory Animals of the National Institutes of Health. The protocol was approved by the Institutional Animal Care and Use Committee of the University of California Irvine (Protocol # 2007–2705). All surgeries were performed under MS222 anesthesia, and all efforts were made to minimize suffering.

**Figure 3:**
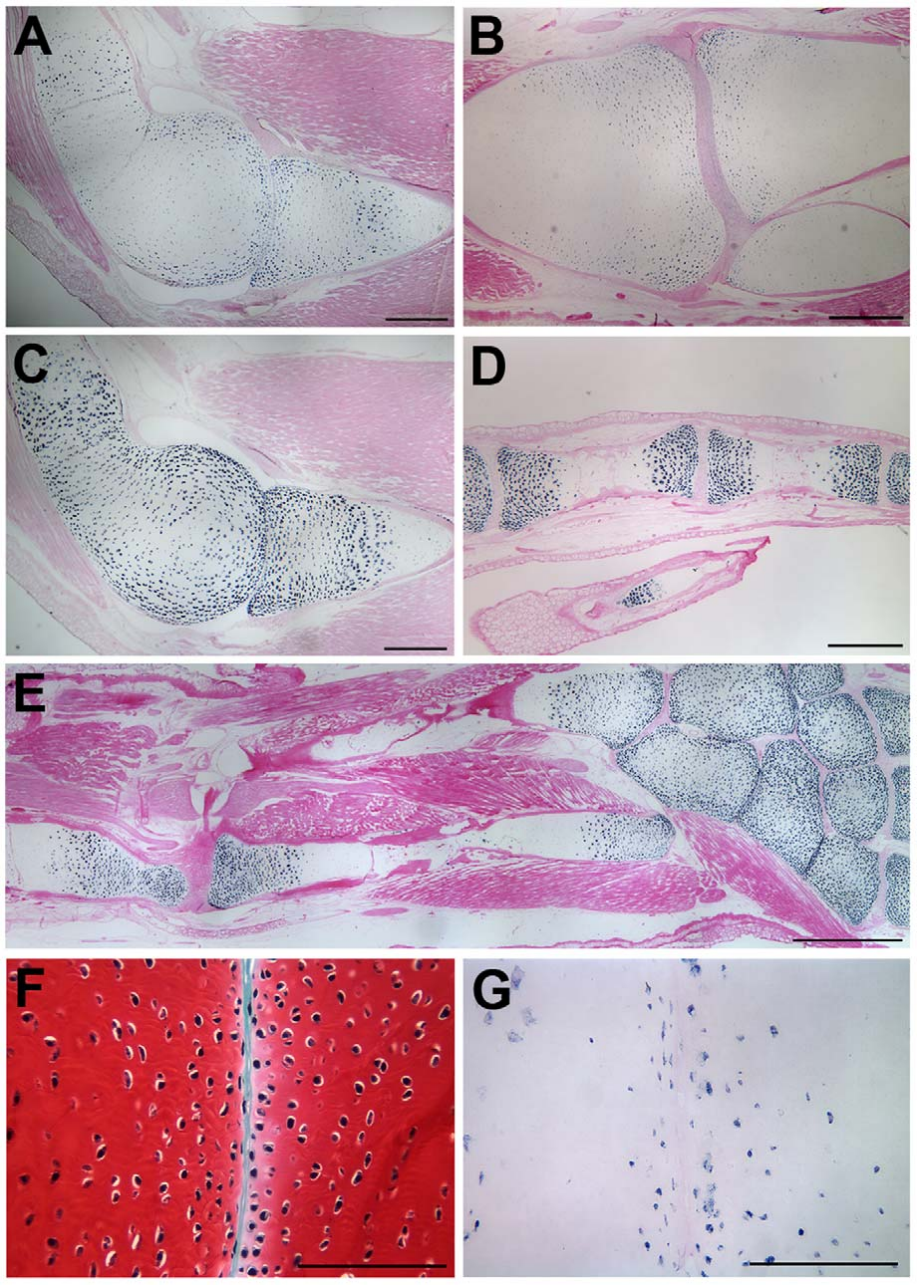
Joint marker gene expression in uninjured axolotl joints. Aggrecan was expressed in the articular cartilage of the epiphysis that was associated with the synovial cavity, as well as at the boundary between the epiphysis and diaphysis. This pattern was the same in both the elbow (A) and the knee (B). Type II-collagen (Col2a1) was expressed throughout the epiphyseal regions of all the joints of the elbow (C), fingers (D), and the hind limb (E), but was not localized to the articular cartilage. CD44 was expressed in cells scattered throughout the epiphyseal cartilage, but was mostly localized to the superficial layers of cells of the articular cartilage associated with the synovial cavity (F, G). Tissue sections for *in situ* hybridization were counterstained with Eosin Y (A–E, G). Tissue section in (F) was stained with Fast Green/Safranin O/Weigert’s Iron Hematoxylin. The conserved patterns of expression were confirmed by analysis of tissues sections from seven different animals. Scale bars in (A–C) = 500 µm; (D, F and G) = 200 µm; (E) = 1 mm.

### Animals and Surgical Procedures

Experiments were performed on axolotls (*Ambystoma mexicanum*) measuring 8–14 cm from snout to tail tip that were spawned at the University of California, Irvine or the Ambystoma Genetic Stock Center at the University of Kentucky. For all surgeries, animals were anesthetized in a 0.1% solution of MS222 (Ethyl 3-aminobenzoate methanesulfonate salt, Sigma), pH 7.0. Animals were kept anesthetized and covered with moist lab tissues for one hour post-surgery. Regeneration was induced by amputation through proximal (mid-humerus or femur) or distal (mid-radius/ulna or tibia/fibula) levels of the limb. For induction of metamorphosis, 10–12 cm axolotls were treated with thyroid hormone (L-Thyroxine, Sigma) according to methods described by Page and Voss [13].

**Figure 4:**
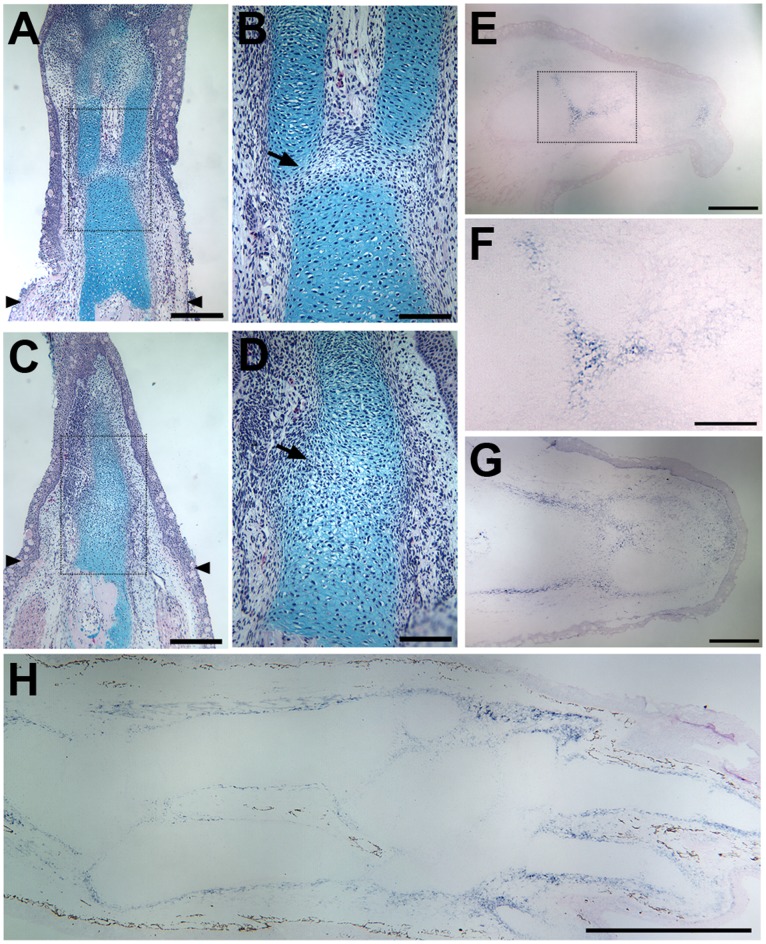
Morphology and patterns of gene expression during elbow and knee regeneration. After an amputated limb had formed a blastema (3 weeks post-amputation), cells in the central region began to condense and differentiate as chondrocytes, at which point they stained with Alcian Blue (Fig. 4A–D). Blastema cells at more distal regions of the blastema (toward the top of Figs. A and C) had not begun to form cartilage condensations. At the same stage of regeneration, joints began to appear at more proximal levels (toward the bottom of Figs. A and C) as the cartilage condensations segregated into discrete skeletal elements with an interzone region (arrows in B, D) that expressed the marker gene *Gdf5* (E, F). Tenascin-C was an early marker (3 weeks post-amputation) for both the perichondrium of the diaphyseal region and the regenerating articular cartilage associated with formation of the interzone (G). Expression of tenascin-C remained high in the perichondrium at later stages of regeneration (5 weeks post-amputation), but decreased in the regions where the joints were regenerated (H). Tissue sections in (A–D) were stained with Alcian Blue/hematoxylin/Eosin Y. Sections for *in situ* hybridization were counterstained with Eosin Y (E–H). Boxed areas in (A) and (C) are illustrated at higher magnification in (B) and (D) respectively. Arrowheads in (A) and (C) indicate the level at which the limbs were amputated. The conserved patterns of expression were confirmed by analysis of tissues sections from five different animals. Scale bars in (A, C, E, and G) = 500 µm; (B, D and F) = 200 µm; (H) = 1 mm.

To create a defect in the proximal epiphysis of the radius or tibia, we made three incisions in the skin overlying the elbow/knee joint area so as to create a skin flap that was still attached to the arm skin on the forth side of the square. We reflected the flap back to expose the underlying soft tissues, and then reflected the muscle fibers to expose the joint. We then used microforceps and iridectomy scissors to dissect the adherent connective tissues, muscle and tendon between the proximal radius/tibia and distal humerus/femur, and removed a 1-mm segment of the epiphysis from the radius/tibia. We then repositioned the soft tissues and the skin flap, which healed into place without sutures by reepithelialization within 6–8 hr [14], [15].

**Figure 5:**
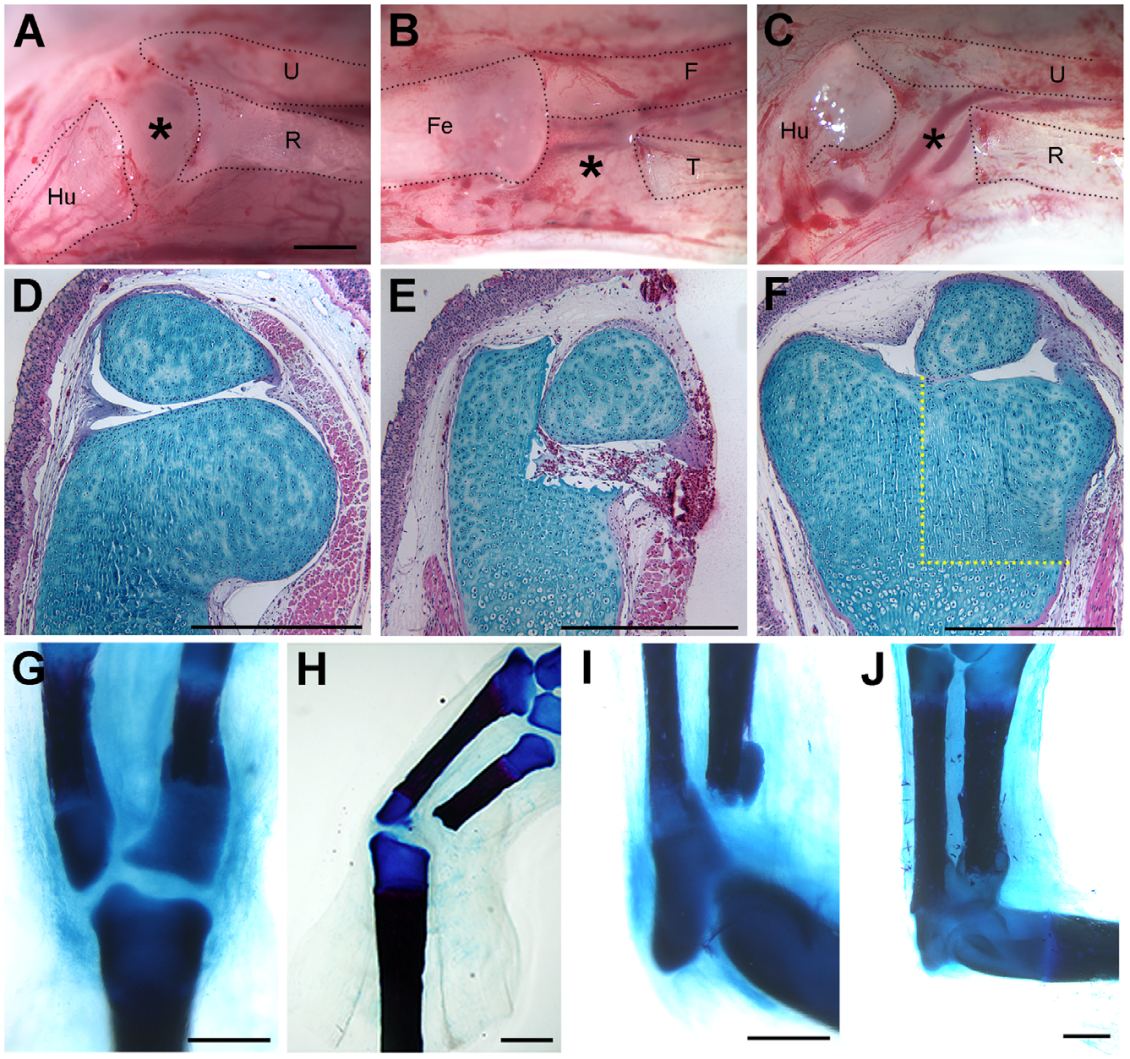
Regeneration of sub-critical size defects in axolotl limb joints. A defect in the distal region of the epiphysis of the humerus (A, D–E) was regenerated (F) within 8 weeks after surgery (macroscopic view of the surgery is illustrated in A). Similarly, a 1 mm defect in the proximal epiphysis of the tibia (surgery illustrated in B) regenerated (G), as did the same size defect in the proximal radius (J; surgery illustrated in C). The regenerative response to excision of 1 mm of the proximal radius was variable. In smaller animals (8–10 cm snout to tail tip), a 1 mm defect failed to regenerate (I); however, in larger animals (12–14 cm), a 1 mm defect was regenerated within 6 weeks post-amputation (J). Defects greater than 1 mm failed to regenerate in either the radius (not illustrated) or the tibia (H). Tissue sections were stained with Alcian blue/Hematoxylin/Eosin Y (D–F). Whole-mount limbs were stained with Alcian Blue/Alizarin Red (G–J). The black dotted lines in A–C demarcate the skeletal elements that remained after the surgical defect was created (Hu, humerus; U, ulna; R, radius; Fe, femur; F, fibula; T, tibia). The yellow dotted lines in (F) indicate the region of the defect that was regenerated (compare to E). Results were confirmed by experimental replication in three animals for distal humerus excisions, seven animals for proximal tibia excisions, and eight animals for proximal radius excisions. The magnification is the same in A–C. Scale bars = 1 mm.

### Histological Analysis

To visualize skeletal elements in whole-mount preparations, we fixed samples in 10% Z-Fix (Anatech) diluted with 40% Holtfreter’s solution overnight. The skin was removed manually after fixation, the samples were refixed in 30% Z-Fix/70% ethanol for 2 hours, and then stained with 0.1% Alizarin Red S in 95% ethanol : 0.3% Alcian Blue 8GX in 70% ethanol : 70% ethanol (1∶1∶8) for 1–2 days at room temperature. Stained tissue samples were treated with 2% KOH to digest the connective tissues to the point where the skeletal tissues could be visualized. The samples were cleared stepwise by soaking in 2% KOH/25% Glycerol, 2% KOH/50% Glycerol, and 2% KOH/75% Glycerol for overnight each, followed by storage in 100% Glycerol.

For staining sectioned samples, collected tissues were fixed in Z-FIX, and treated with Decalcifier I (Surgipath),to decalcify the bones of the limb tissues in order to facilitate subsequent sectioning. Tissues were then dehydrated with a ascending series of ethanol (25%, 50%, 75% and 100%), cleared in xylene, embedded in paraplast, and sectioned at 6 µm thickness. For Alcian Blue staining, the sectioned samples were stained with a solution of 0.03% Alcian blue/0.1% HCl/70% ethanol for 30 min, followed by standard hematoxylin and eosin Y staining. For Fast Green/Safranin O staining, the sectioned samples were stained with 1% Fast Green and 0.1% Safranin O, followed by Weigert’s Iron Hematoxylin Solution.

### Cloning of the Coding Region of Axolotl CD44

We used sequence and homology data from the *Ambystoma* EST database for cloning and generating probes for target axolotl genes. Homologies to the human RefSeq database were annotated as “best hit” with a BLASTX threshold of *E* = 10−7 (www.ambystoma.org). To obtain full-length coding sequence for the axolotl ortholog of *CD44*, 5′ and 3′ mRNA sequences that contain the start and stop codons of axolotl *CD44* were retrieved from the *Ambystoma* EST Database (ID#: 5′- C100076, 3′-C261340). Axolotl *CD44*-specific primers were designed based on the EST sequences to amplify the intervening sequence by RT-PCR. Total RNA from axolotl larvae was isolated using miRNeasy Mini Kit (Qiagen) following the manufacturer’s recommended protocol. To synthesize first-strand cDNA from the isolated total RNA, Oligo(dT)12–18 primer and SuperScriptIII reverse transcriptase (Invitrogen) were used. Polymerase chain reaction (PCR) was performed using ExTaq DNA polymerase (Takara).with the axolotl *CD44* specific primers: CD44-F; 5′-AACTTCCAGCTAACTCTGCCTG-3′, CD44-R; 5′-CTTTAAGTTCCAGTCCCAGTCC-3′. The full-length coding sequence of axolotl CD44 has been submitted to Genbank, accession # JX457476.

### RNA *in situ* Hybridization (*Aggrecan, CD44, Col2a1, GDF-5, Tenascin-C*)

RNA *in situ* hybridization was performed on paraffin-sectioned axolotl limb tissues. Digoxigenin (DIG)-labeled antisense RNA probes for axolotl *Aggrecan* (*Ambystoma* EST database gene ID#: C065974; 900 bp), *CD44* (1082 bp), *Col2a1* (ID#: C081592; 944 bp), *GDF-5* (ID#: C030457 and ID#: C733258; 1203 bp), and *Tenascin* (ID#: C064822; 905 bp) were used to perform *in situ* hybridization. RT-PCR was performed to amplify the sequences for antisense RNA probes with gene specific PCR primers. The specific PCR primers for the individual genes were as follows:

*Aggrecan* forward: 5′-GATATGCGAAGAAGGATGGACC-3′

*Aggrecan* reverse: 5′-GTCTTCTTCGTTCTTCCCTTGG-3′

*CD44* forward: 5′-AACTTCCAGCTAACTCTGCCTG-3′

*CD44* reverse: 5′-CTTTAAGTTCCAGTCCCAGTCC-3′

*Col2a1* forward: 5′-CACCTATGGATATTGGTGGAGC-3′

*Col2a1* reverse: 5′-GTACATCATCCACTTGGCTACC-3′

*GDF-5* forward: 5′-GTCAACGTGCACGCAGATTCTA-3′

*GDF-5* reverse: 5′-ATTAGGTTGGGTTCCATCCCG-3′

*Tenascin-C* forward: 5′-TACTGGGCTCTACACCATCTAC-3′

*Tenascin-C* reverse: 5′-CCAAGAGGATGACAAGTCTGTG-3′

All template clones were subcloned into the pCRII vector (Invitrogen). To synthesize antisense RNA probes for each of the genes, the plasmid DNA templates were linearized by *BamHI* (for *Col2a1* and *GDF-5*) or *XhoI* (for *Aggrecan, CD44, Tenascin*). The RNA probes were synthesized with the DIG RNA Labeling Kit (Roche) according to the manufacturer’s protocol using T7 (*Col2a1, GDF-5*) or Sp6 (*Aggrecan, CD44, Tenascin*) RNA polymerases. For tissue sample collection, tissues were collected and fixed in MEMFA (0.1M MOPS, pH7.4, 2 mM EGTA, 1 mM MgSO_4_, 3.7% formaldehyde), skeletal tissues were decalcified in Decalcifier I, dehydrated with a ascending series of ethanol (25%, 50%, 75% and 100%), cleared in xylene, and embedded in paraplast. Paraffin sections were cut at 6 µm thickness. Sections were treated with 7.5 µg/ml of Proteinase K (Invitrogen) for 20 min at 37°C, refixed with 4% paraformaldehyde, and then hybridized with antisense RNA probes at 60°C overnight. After hybridization, the section were washed with the buffer #1 (Formamide: water: 20X SSC = 2∶1∶1), and buffer #2 (5∶4∶1), and blocked with 2% blocking reagent (Roche) in TBST for 30 min. The sections were then incubated with 1∶2,000 diluted alkaline phosphatase (AP)-conjugated anti-DIG antibody (Roche) overnight at 4°C. The color staining reaction was performed using BM purple (Roche) as a substrate for AP.

## Results

### Morphology and Gene Expression of Uninjured Limb Joints of the Axolotl

As reported previously [4], [9], the basic anatomy of axolotl joints with apposed articular surfaces between adjacent long bones that are encapsulated by connective tissues was very similar to mammals (Fig. 1). The axolotl elbow joint was most similar to the equivalent mammalian joint in that the synovial space between the apposed skeletal elements was fluid-filled and acellular (Fig. 1A, B). In contrast, the synovial space of the axolotl knee joint was filled by a dense fibro-cellular tissue (Fig. 1C, D), as reported previously [4]. Based on the initial observations of the knee joint [4], these synovial cells were suggested to be equivalent to interzone cells (a developmentally transient population of joint-forming cells in the limb bud) that persisted as a consequence of the neotenous mode of development of the axolotl. Although the axolotl progresses through metamorphosis to the point of developing appendages and becomes sexually mature (neoteny), it does not complete metamorphosis and become terrestrial as occurs in most other salamanders. Therefore it has been hypothesized that the persistence of “interzone-like” cells in the knee joint is a consequence of this arrested development, and that these cells maintain chondrogenic potential and contribute to the repair of joint defects [4], [9].

In order to explore the relationship between the presence and absence of synovial cell and the ability to regenerate joint structures, we examined all the joints in both the axolotl forelimb and hind limb to determine the variability in morphology, and the distribution of acellular and fibro-cellular synovial joints. As reported recently [9], we observed that both types of joint morphologies were present in both forelimbs and hind limbs (Fig. 1). In general, there was a trend with the proximal joints being acellular and more distal joints being fibro-cellular. Thus the shoulder and elbow joints were acellular (Fig. 1A, B; not illustrated for the shoulder joint), and the wrist (Fig. 1E) and interphalangeal joints (Fig. 1F) were fibro-cellular. Although many of the joints between the carpals in the wrist were fibro-cellular, some of the articulations between the distal zeugopod and proximal autopod were acellular (Fig. 1E, arrow), and thus the wrist joints exhibited an intermediate phenotype. In the hind limb, the hip joint was acellular (Fig. 1G); whereas, the knee (Fig. 1C, D) and interphalangeal joints (same morphology as in the forelimb, Fig. 1F) were fibro-cellular. As observed in the wrist region, the joints of the ankle were a mixed phenotype of both acellular and fibro-cellular (Fig. 1H, arrow) even though the knee joint was fibro-cellular. Thus the presence of mixed phenotype joints was associated with the boundary between the zeugopod and autopod, rather than being a transitional phenotype between acellular and fibro-cellular joints along the proximal-distal limb axis. Since all the joints in both the forelimb and hind limb regenerate when the limb is amputated, there does not appear to be a relationship between joint regeneration and the presence or absence of interzone-like cells.

In order to test whether the persistence of interzone-like cells is a consequence of neoteny in axolotls, and whether they are unique to axolotl limbs, we examined the morphology of limb joints in axolotls that had undergone metamorphosis, as well as the joints in a post-metamorphic frog (*Xenopus tropicalis*) (Fig. 2). Although axolotls typically do not complete metamorphosis, individuals will occasionally undergo metamorphosis spontaneously. Experimentally, metamorphosis can be induced by adding thyroid hormone to the aquarium water [13]. The morphology of both the elbow (Fig. 1A, B and Fig. 2A, B) and knee joints (Fig. 1C, D and Fig. 2C, D) were the same in both pre-metamorphic axolotls (Fig. 1) and post-metamorphic axolotls (Fig. 2). The same pattern of joint morphology observed in the axolotl was present in post-metamorphic frog limb joints (acellular elbow joint, Fig. 2E and fibro-cellular knee joint, Fig. 2F). Thus the persistence of interzone-like cells is not related to metamorphosis, and is not a novel feature of axolotl joints.

In addition to a conserved morphology, uninjured limb joints in the axolotl expressed a number of joint marker genes in spatial patterns that were comparable to what is observed in mammalian joints (see [5]). The proteoglycan, Aggrecan was expressed in the articular cartilage of the epiphysis that was associated with the synovial cavity, as well as at the boundary between the epiphysis and diaphysis (Fig. 3A, B). This pattern was the same in both the elbow (Fig. 3A) and the knee (Fig. 3B). Type II-collagen (Col2a1) expression was localized to the epiphyseal regions of all the joints of both the forelimb (Fig. 3C, D) and hind limb (Fig. 3E). Finally, CD44 was expressed in cells scattered throughout the epiphyseal cartilage, but was mostly localized to the superficial layers of cells of the articular cartilage associated with the synovial cavity (Fig. 3F, G). Previous studies of the fibro-cellular knee joint reported similar but not identical expression patterns for Aggrecan and Type II-collagen proteins [4], [9]. Our *in situ* hybridization data provide validation of the specificity of the heterologous antibodies used for immunohistochemistry in those studies. In addition, Type I-collagen, GDF5 and BOC (Brother of CDO) were reported to be expressed in association with the fibro-cellular tissues of the uninjured knee joint [4], [9].

### Morphology and Gene Expression of Regenerating Limb Joints in the Axolotl

The events associated with the regeneration of joints of amputated limbs appeared to be the same as occur during limb development (see [5]). During the later stages of blastema growth, cells in the central region began to condense and differentiate as chondrocytes, at which point they stained with alcian blue (Fig. 4A–D). As the blastema continued to grow distally, joints began to form more proximally as chondrogenic cells began to form an interzone as evidenced by the expression of *Gdf5* (Fig. 4E, F). *Gdf5* expression was transient in joints that subsequently underwent cavitation to form an acellular synovial cavity (e.g. elbow). In contrast, *Gdf5* expression was reported to persist in joint that have fibro-cellular tissue within the synovial cavity (e.g. knee; [9]). Tenascin-C was an early marker for both the perichondrium of the diaphyseal region and the regenerating articular cartilage associated with formation of the interzone (Fig. 4G). Expression of tenascin-C remained high in the perichondrium at later stages of regeneration, but decreased in the regions where the joints were regenerated (Fig. 4H). In addition, the expression patterns of the joint marker genes described above for uninjured joints (Fig. 3) were reestablished by the end of regeneration.

### The Regenerative Response to Injury was Dependent on the Extent of the Defect

We determined that the ability of the axolotl to regenerate surgical defects to articular cartilage of joints was dependent on the size of the defect, and to some extent the size and age of the animal. We confirmed the previous report that a defect in the femoral condyle was repaired endogenously (data not shown; [4]). This intrinsic regenerative response was not restricted to the knee joint that has fibro-cellular tissue, but also occurred in the elbow joint that has an acellular synovial cavity (Fig. 5A, D–F). A resection of the distal humerus comparable to what was reported previously for the distal femur [4], also regenerated endogenously Fig. 5F). Similarly, a 1 mm defect in the proximal epiphysis of the tibia regenerated (Fig. 5B, G), as did the same size defect in the proximal radius (Fig. 5C, J). Thus the bones that were apposed in the knee and elbow joints responded the same. In our initial experiments with smaller animals (8–10 cm snout to tail tip), a small (1 mm) defect in the proximal radius failed to regenerate (Fig. 5I; n = 10 animals); however, when we repeated this experiment with larger animals (12–14 cm), nearly all the 1 mm defects were regenerated (Fig. 5J; n = 5 of 6 animals). Thus the ability to regenerate a small defect in the articular cartilage of the epiphysis was not related to the presence or absence of interzone-like cells in the synovial cavity.

The intrinsic ability of the axolotl to regenerate joint defects was limited to relatively small defects. For both the forelimb and hind limb, defects that were greater than 1 mm failed to regenerate (e.g. Fig. 5H; n = 3 forelimb; n = 3 hind limb). Thus the response to joint injuries in the axolotl was comparable to what occurs when defects were created in the diaphyseal region in both the axolotl and in mammals [11], [12], [16]. Defects heal when they are smaller than a critical size (Critical Size Defect, CSD), but form a callus and fail to regenerate when greater than the CSD. In the axolotl, a diaphyseal defect that exceeds a CSD can be induced to regenerate in response to signaling from a deviated nerve and wound epithelium [12]. In spite of attempts to induce regeneration of proximal radial defects with a deviated nerve and wound epithelium (n = 4 animals), we did not observe evidence of an enhanced regenerative response relative to control defects (Fig. 5I).

## Discussion

The anatomy of limb joints in the axolotl, particularly the acellular joints such as the elbow is conserved compared to mammals and other tetrapods. Similarly, the expression patterns of marker genes in both the uninjured and regenerating axolotl joints are comparable to what is observed in uninjured and developing tetrapod limbs. In addition, the sequence of events during joint regeneration leading to interzone formation and cavitation is the same as during limb development. Taken together, these findings suggest that a similar molecular mechanism is used during the development of many joints [17], and this same mechanism is used again during regeneration of limbs and joints [18].

The presence of fibro-cellular tissues in some of the axolotl joints has raised the question of the functional relationship of these interzone-like cells to regeneration [4], [9]. At this point, it appears that interzone cells are neither necessary nor sufficient for joint regeneration. These cells are not necessary for joint regeneration in an amputated limb since all the different limb joints are regenerated perfectly whether or not they have interzone-like cells. These cells also are not sufficient for regeneration of a joint defect in the absence of limb amputation. Both the elbow and knee joints regenerate a sub-critical size defect, and neither can regenerate a defect greater than the CSD, even though interzone-like cells are present in the knee joint. Finally, both acellular and fibro-cellular joints are present in post-metamorphic *Xenopus* froglets that do not regenerate any joints when a limb is amputated [19], [20].

Although interzone-like cells may not be directly involved in an endogenous regenerative response, they may have the potential to be a source of cells for the repair of skeletal tissues. When grafted into a non-regenerating defect in the diaphysis they participate in the formation of an ectopic joint-like structure (pseudarthrosis) and contribute both to the chondrocytes at the ends of the skeletal elements and the intervening fibro-cellular tissue [9]. A major challenge in the field of regeneration biology is understanding how specific progenitor cell types are recruited to participate in regeneration. It has been difficult to address this issue given the diversity of cell types involved and the lack of molecular markers for regeneration-competent cells. The homogeneity of axolotl interzone-like cells and the markers that we have validated provide the opportunity to test this population of cells for the ability to respond to regeneration-inducing signals [12], [21] and function as a multipotent progenitor cell for the repair and regeneration of joints in response to both acute and chronic damage.
